# Novel Muscle in Infraspinous Fossa

**DOI:** 10.7759/cureus.56100

**Published:** 2024-03-13

**Authors:** Emma R Lesser, Chung Yoh Kim, Keishiro Kikuchi, Samir Anadkat, Joe Iwanaga, R. Shane Tubbs

**Affiliations:** 1 Department of Neurosurgery, Center for Clinical Neurosciences, Tulane University School of Medicine, New Orleans, USA; 2 Department of Orthopaedics, Kurume University School of Medicine, Fukuoka, JPN; 3 Departments of Anatomy and Structural and Cellular Biology, Tulane University School of Medicine, New Orleans, USA; 4 Department of Anatomical Sciences, St. George's University, St. George's, GRD; 5 Department of Neurosurgery, Ochsner Neuroscience Institute, Ochsner Health System, New Orleans, USA

**Keywords:** cadaver, shoulder, variations, deltoid muscle, infraspinatus muscle

## Abstract

The infraspinatus muscle (IS) makes a minor contribution to lateral rotation of the arm but mainly serves to stabilize the glenohumeral (GH) joint as part of the rotator cuff. Although reports of variations in the rotator cuff muscles have been documented previously, specific discussions of IS variants are lacking. In this report, we present a novel case of an accessory muscle in the infraspinous fossa and its relationship to the IS, which was normally located. We describe the observed physical features of the muscles and their innervation patterns.

## Introduction

Muscles in the scapular region are pivotal in moving the upper limbs around the glenohumeral (GH) joint. Most of these muscles have proximal attachments in the scapula and clavicle and distal attachments in the humerus, enabling the upper limbs to engage in a wide range of movements around the ball and socket GH joint. 

Four muscles (supraspinatus (SuS), infraspinatus (IS), teres minor (Tm), and subscapularis (SbS)) in this region are called rotator cuff muscles because they form a musculotendinous cuff around the GH joint at the most proximal end of the humerus. As part of the rotator cuff and because it is so close to the GH joint's axis of rotation, the IS is among the muscles principally responsible for the stabilization of the GH joint while it undergoes movements that may be brought on by the surrounding muscles [1]. The IS and other rotator cuff muscles contribute to stabilizing and protecting the joint by fusing with fibrous layers of the joint capsule [1]. Injuries or pathological lesions of the rotator cuff muscles harm the movements and stability of the joint. 

The deltoid muscle (DELT), another major controller of GH joint movements, has a triangular or delta shape covering the shoulder [1]. Its rounded proximal attachments enable multiple movements of the GH joints to be made, but its main action is abduction of the arm. Although the DELT is a powerful abductor, the SuS initiates the first 15 degrees of abduction of the limb [1].

Differences in typical musculature can be crucial both anatomically and clinically because variations, such as additional heads, can present unforeseen surgical obstacles. Reports of such variant anatomy increase awareness of potential surgical complications and allow for anatomical terminology to improve and reflect the anatomy that is seen presently [2]. In this paper, we report a rare and previously undefined muscle in the infraspinous fossa area and discuss the potential embryological origins of this variation.

## Case presentation

**Figure 1:**
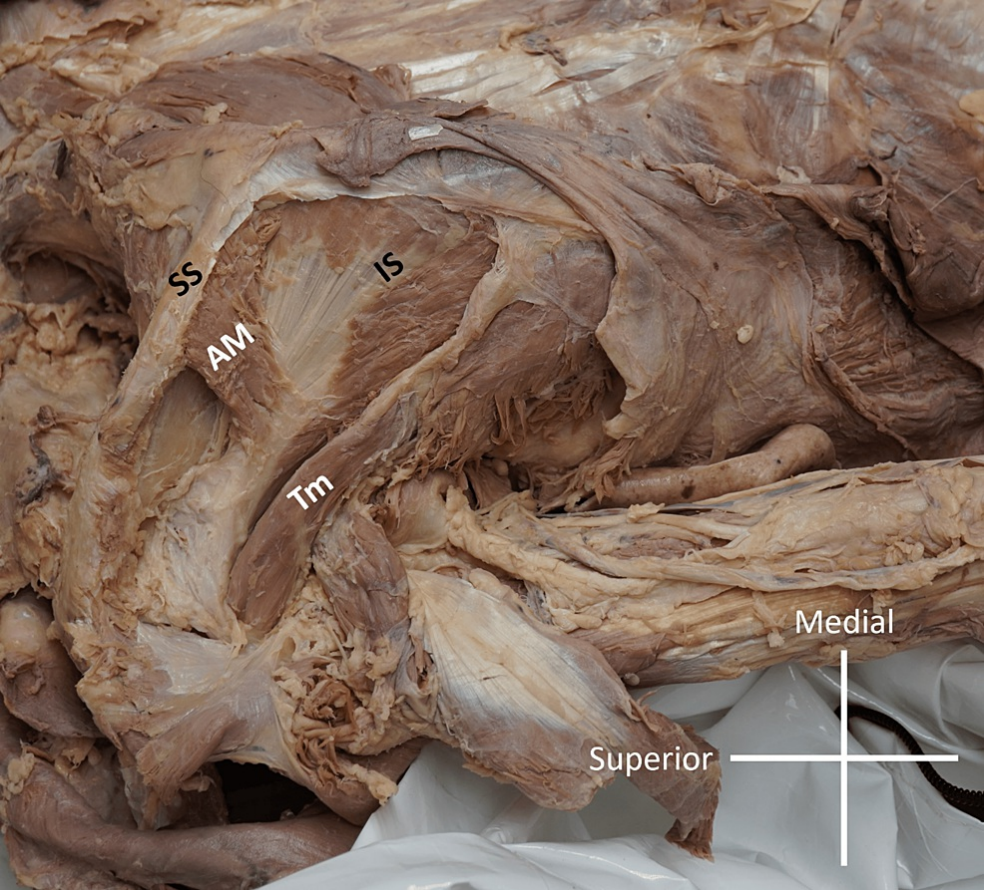
Low magnification view of left scapula TM: teres minor muscle; IS: infraspinatus muscle; AM: accessory muscle; SS: spine of scapula

**Figure 2:**
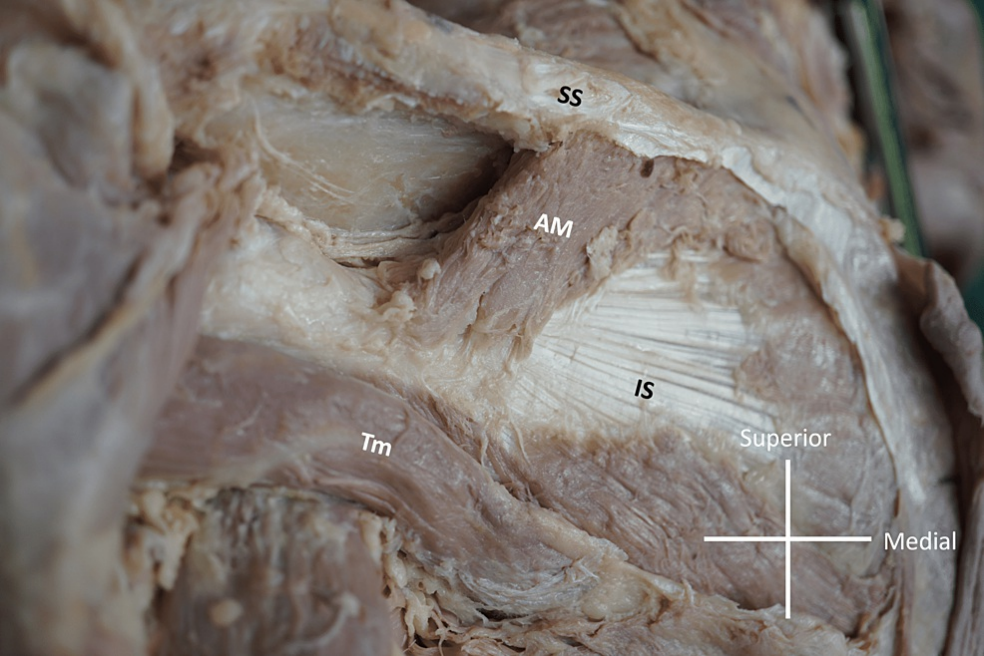
Accessory muscle seen with spine of scapula The accessory muscle (AM) is seen originating from the inferior lip of the spine of the left scapula (SS) and inserting into the tendon of the infraspinatus muscle (IS). For context, the teres minor muscle (Tm) has been identified.

During routine dissection of the scapular region, an accessory muscle was observed in the infraspinous fossa of the left scapula (Figure 1). The donor was female and 87 years old at death and died of complications related to chronic obstructive pulmonary disease. The fibers of this accessory muscle were positioned orthogonally to those of the IS (Figure 2).

Superficial to the IS, which appeared normal, the additional muscle (AM) stretched from the spine of the scapula inferoposteriorly to the tendon of the IS. Its dimensions were measured using a digital microcaliper (Mitutoyo, Kanagawa, Japan). It was 27.08 mm long, 20.93 mm wide and 4.78 mm thick. It was noted that the fiber direction of the AM followed the direction of the deltoid muscle that lay at the most superficial muscular layer of the shoulder region.

**Figure 3:**
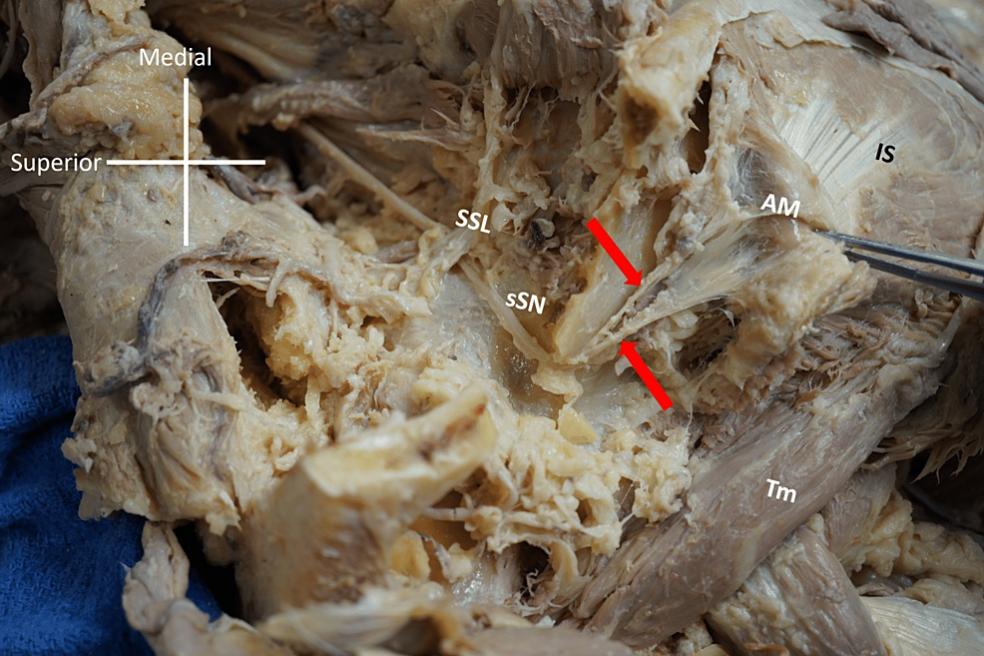
Accessory muscle with spine of scapula resected Spine of scapula resected to show suprascapular nerve (sSN) traveling freely and uncompressed under suprascapular ligament (SSL) and coursing to the posterior surface of the scapula and is seen giving branches (red arrows) to supply the accessory muscle (AM) (which has been reflected). TM: teres minor muscle; IS: infraspinatus muscle

The IS on the contralateral (right) side was atrophied, but less severely than that on the left. Comparatively, the left IS had a more robust muscle tendon whereas the muscle belly appeared to be atrophied upon gross inspection. The spine of the scapula was then resected using an osteotome and mallet. Removal of the bony structure enabled the innervation of the accessory muscle to be elucidated. Like the other muscles on the posterior face of the scapula, it received branches from the suprascapular nerve (sSN) (Figure 3).

The present study of the cadaveric specimen was performed in accordance with the requirements of the Declaration of Helsinki (64th WMA General Assembly, Fortaleza, Brazil, October 2013). The authors state that every effort was made to follow all local and international ethical guidelines and laws that pertain to the use of human cadaveric donors in anatomical research [3].

## Discussion

The perpendicular nature of the muscle fibers suggests that a possible function could be to tug on, or elevate, the IS, altering the direction of the force applied by the IS. The location of the additional muscle leads us to suggest that it could aptly be classified as a “levator infraspinatus muscle.” Since it seems to have received innervation from the sSN, it is likely to be a variant of one of the muscles already innervated by this nerve. The fiber direction is similar to that of the deltoid muscle, but the discrepancy in innervation suggests that the variant in question is derived from the IS rather than the DELT [1]. 

The accessory muscle is particularly interesting because of its placement on the scapula and its relationship to the underlying IS. It travels only a few millimeters and connects bone (i.e., the spine of the scapula) to muscle (i.e., the tendon of the IS). Perhaps the perpendicular fibers adjusting the magnitude of the contractile force of the IS partly explain why the ipsilateral IS was severely atrophied in relation to right IS. The extra muscle could be responsible for movements that the IS would ordinally accomplish unassisted, thereby decreasing the mechanical demands on the IS. As such, perhaps the presence of the accessory muscle compensated for a weaker rotator cuff which may have been clinically noticeable otherwise. However, it is worth noting that the resulting deficiency from a weak IS would be minimal as the IS is one in a group of several muscles that perform a common function.

Variations of muscles in the scapular region are not well reported. Much of the literature on variations of the IS dates back to the 19th century and has not been revisited since 2016 [4-7]. Macalister [5] describes classes of muscular variations in his “Notes on Muscular Anomalies in Human Anatomy”, two of which may pertain to the variation seen here. In Class I, he describes anomalies resulting from muscles that are occasionally present, proposing that they are likely to be remnants of vestigial muscles. This category contains an example of a variant of the IS, the IS secundus (secIS), but it was described as deep to the subscapularis on the subscapular fossa of the scapula. The secIS is oriented differently from the muscle observed in our case and was ruled out as a possible identification. 

The second group of muscular variants includes muscles with duplicates in planes orthogonal to that of the primary muscle [5]. This group seems to be closest to describing the accessory muscle in our case, but the IS has not been mentioned as an example of such a variation. 

In Class III, typically seen muscles have an extra origin and/or more tendons than usual [5]. Perhaps the muscle that was observed here is another head and tendon of the IS. Within this third grouping, Macalister [6] introduces a muscle called the “scapulo-humeralis digastricus singularis of Gruber” (SHDs) as a possible variant. The German paper [7] in which the SHDs was first reported describes it as having two origins with a common insertion on the humerus. One of the origins matches the muscle observed in our specimen, but the attachment differs; the observed muscle stretches from the spine of the scapula inferiorly to the tendon of the IS. 

Embryology

There are two possible assumptions for classifying this muscle: it is another variant of the IS, or it is a variant of the deltoid. Both the IS and DELT originate from a common premuscle mass during development. In the 11 mm embryo, development of the acromion partially separates the supraspinatus and IS, and the DELT partially splits off from the mass towards its origin from the acromion and clavicle [8,9]. The most reasonable argument for considering the muscle to be an IS variant is its innervation from the sSN. During the dissection, the suprascapular nerve was confirmed by passing the suprascapular notch in the anterior side of the scapula and the greater scapular notch (spinoglenoid notch) (as seen in Figure 2). After passing the latter, the nerve innervated the muscle and the IS. However, in terms of shape, the muscle was directed orthogonally to the IS, and its direction also differed from other previously reported variations of the IS [10-12]. Rather, the direction was similar to that of the posterior part of the DELT. Considering that both the suprascapular and axillary nerves have fifth and sixth cervical nerve components, it is not unreasonable to regard the muscle as a variant of the deltoid. Further studies are required to determine the nature of this muscle variation.

The authors sincerely thank those who donated their bodies to science so that anatomical research could be performed. Results from such research can potentially increase mankind’s overall knowledge, which can then improve patient care. Therefore, these donors and their families deserve our highest gratitude [13]. 

## Conclusions

This variant and a review of the variations in the scapular region musculature could provide insight into functional disorders associated with a rotator cuff injury. Additionally, knowledge of variations that influence changes in normal muscle morphology can be screened for in clinic, and more can be learned about the possible functional roles these abnormalities could possess.

## Appendices

Acknowledgement

The authors sincerely thank those who donated their bodies to science so that anatomical research could be performed. Results from such research can potentially increase mankind’s overall knowledge that can then improve patient care. Therefore, these donors and their families deserve our highest gratitude [13]. 
